# A Neural Network Based Intelligent Predictive Sensor for Cloudiness, Solar Radiation and Air Temperature

**DOI:** 10.3390/s121115750

**Published:** 2012-11-12

**Authors:** Pedro M. Ferreira, João M. Gomes, Igor A. C. Martins, António E. Ruano

**Affiliations:** 1 Algarve Science and Technology Park, Campus de Gambelas, Pav. A5, 8005-139 Faro, Portugal; 2 Centre for Intelligent Systems, IDMEC-IST, Av. Rovisco Pais 1, 1049-001 Lisboa, Portugal; E-Mail: aruano@ualg.pt; 3 Department of Electronic and Informatics Engineering, University of Algarve, 8005-139, Faro, Portugal; E-Mails: joaomealhagomes@gmail.com (J.M.G.); rogimartins13@gmail.com (I.A.C.M.)

**Keywords:** intelligent sensor, sensor fusion, neural networks, cloudiness estimation, solar radiation prediction, temperature prediction, genetic algorithms

## Abstract

Accurate measurements of global solar radiation and atmospheric temperature, as well as the availability of the predictions of their evolution over time, are important for different areas of applications, such as agriculture, renewable energy and energy management, or thermal comfort in buildings. For this reason, an intelligent, light-weight and portable sensor was developed, using artificial neural network models as the time-series predictor mechanisms. These have been identified with the aid of a procedure based on the multi-objective genetic algorithm. As cloudiness is the most significant factor affecting the solar radiation reaching a particular location on the Earth surface, it has great impact on the performance of predictive solar radiation models for that location. This work also represents one step towards the improvement of such models by using ground-to-sky hemispherical colour digital images as a means to estimate cloudiness by the fraction of visible sky corresponding to clouds and to clear sky. The implementation of predictive models in the prototype has been validated and the system is able to function reliably, providing measurements and four-hour forecasts of cloudiness, solar radiation and air temperature.

## 1. Introduction

Nowadays there is a global need for decreasing our energy consumption, to ensure that what we get from our natural resources is efficiently used, and that we live in balance with the environment. Increasingly so, efforts in research and development are directed towards this goal. A major field of research consists in developing intelligent systems capable of integrating environmental data, to improve efficiency in the utilisation of resources and to enable sustainable functioning of man-made utilities. In this study, the main focus is on global solar radiation, as it influences the majority of living beings in many different ways. Thus, an accurate prediction of its evolution over time is important for several different areas of application, such as agriculture, renewable energy, or people's comfort in buildings, where possible applications are luminescence control and thermal comfort. For the last case, the authors have successfully employed predictive models of solar radiation, air temperature and relative humidity in order to improve the optimisation of energy consumption by Heating Ventilation and Air Conditioning devices and to maintain thermal comfort in public buildings [1–3].

In the field of meteorological data acquisition and estimation, more specifically on cloudiness, solar radiation and atmospheric temperature, there is room for further developments. Firstly, there are not many different systems which incorporate, as a package, the acquisition of sky pictures and sensor data. Secondly, the existing commercial solutions that employ sky images for academic cloudiness studies are not portable in a way to allow easy assembling on the spot. Finally, and more importantly, no portable commercial solution incorporates the possibility of forecasting the estimated or measured variables.

Although over the last decade intelligent sensors have been researched and implemented, the focus has been on extending functionality by using Artificial Neural Networks (ANNs) to perform some complex data processing. Examples are the linearisation of sensor outputs [4], the compensation of non-linearities induced by environmental quantities [4–7], the fusion of multiple sensors data into one or more improved sensor outputs [8,9], or the classification of pre-processed sensor data [10,11]. Besides functionality extension, the efficiency of intelligent sensors has also been addressed. Examples are the extension of self-powered wireless sensor nodes' battery lifetime using artificial learning methodologies [12], neural network based auto-calibration procedures [13], or the development of a system-on-chip device to ease the implementation and standardisation of intelligent sensors [14].

This work introduces the use of ANNs as time-series predictors that enable sensor forecasting outputs in addition to the typical measurement outputs available in common sensors for solar radiation [15] and air temperature [16]. For this purpose Radial Basis Function (RBF) ANNs have been used as one-step-ahead predictors, which are then iterated in a multi-step fashion to yield forecasts in larger horizons. The RBF predictive models have been identified using a system identification framework based on a Multi-Objective Evolutionary Algorithm (MOEA) [17–19]. This approach not only estimates the model parameters, but also determines the network topology and performs input selection.

The prototype device implemented is a product of previous work by the authors on the prediction of global solar radiation [20–22] and on the estimation of cloudiness [22–24], which in turn was motivated by applications on the energy efficiency and thermal comfort in buildings [2,3,25–28], and on the agricultural field [29–31].

**Figure 1.**
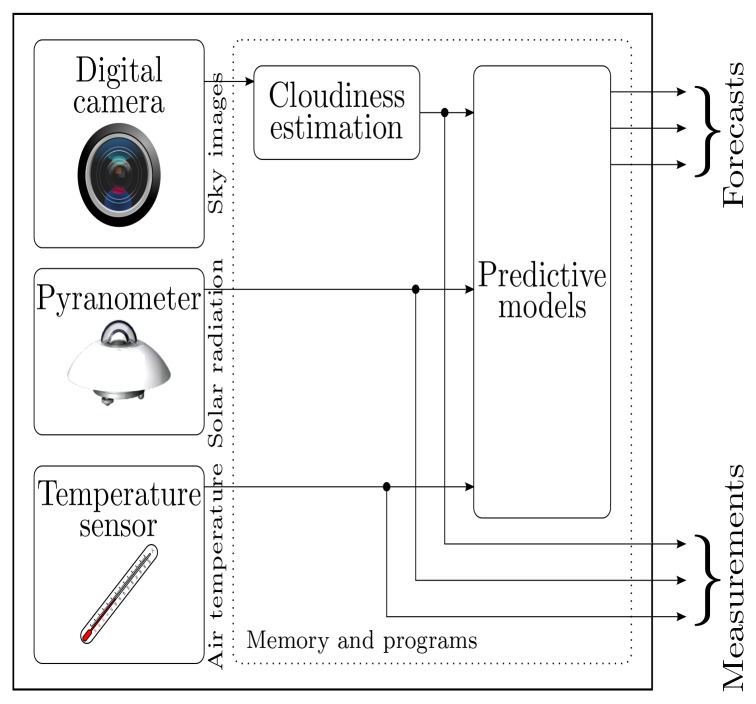
Illustration of required functionalities of the device.

The development of the intelligent sensor prototype was motivated by the requirement of integrating data acquisition, variable estimation, and prediction functionalities into a single device, as illustrated in Figure 1. By using such system, variable measurements and the corresponding forecasts may be stored in memory and made available to external systems by means of common networking technologies, such as Ethernet or Wi-Fi, and, for instance, network sockets. Regarding cloudiness, the current implementation considers only daytime operation, from dusk to dawn, providing 0% cloudiness measurements/predictions during night-time periods. Another temporary limitation is a decreased prediction performance arising from the fact that the thresholding ANN employed in cloudiness estimation was trained with images acquired by a different system. This has a negative impact in the amount of cloudiness estimated, which then propagates to the remaining models. Acquiring a data set of images with the prototype covering different seasons of the year, different weather conditions and different cloud cover situations requires a whole year only to collect images. This work in progress will enable the training of an ANN with images acquired by the prototype, therefore resolving the current limitation. The option, presented in this paper, was to completely develop the device and validate its implementation, both software and hardware.

The paper is organised as follows. As the cloudiness estimates and the various forecasts are obtained by means of RBF ANNs, Section 2 describes the model identification methodologies. These are used in order to identify an image segmentation model for cloudiness estimation (Section 3) and the predictive models for global solar radiation and air temperature (Section 4). The prototype intelligent sensor is described in detail in Section 5. Finally, Section 6 presents results considering the utilisation and validation of the system.

## 2. Model Identification

The RBF ANN models are trained by the Levenberg–Marquardt (LM) algorithm [32,33] using a modified training criterion [34,35], and the model structure (number of neurons and input terms) is selected by a procedure using a Multi-objective Genetic Algorithm (MOGA) [36].

Although a detailed and complete description of the model identification methodology is beyond the scope of this article, the following subsections give enough detail to enable full understanding on the identification of the models used in the prototype device. For further information the interested reader should consult the previous work [17–19,37].

### 2.1. RBF Parameters Determination

The RBF ANNs have the form,

(1) $$\hat{y}(\text{x},\text{w},\text{C},\text{σ})=\sum_{i=0}^{n}w_{i}\varphi_{i}(\text{x},{\text{c}}_{i},\sigma_{i})$$

where typically *φ_i_* is the Gaussian function,

(2) $$\varphi_{i}(\text{x},{\text{c}}_{i},\sigma_{i})=e^{-\frac{1}{2\sigma_{i}^{2}}{\left\|x-c_{i}\right\|}^{2}},\varphi_{0}=1$$

For a specified number of neurons, n, and for a determined set of inputs, **X***^t^*, off-line training a RBF ANN corresponds to determining the values of **w**, **C**, and ***σ*** such that Equation (3) is minimised:

(3) $$\Phi ({\text{X}}^{t},\text{w},\text{C},\text{σ})=\frac{1}{2}{\left\|\text{y}-\hat{\text{y}}({\text{X}}^{t},\text{w},\text{C},\text{σ})\right\|}^{2}$$

Please note that Equation (3) is now applied to a set of training input patterns, **X***^t^*, and not to a single input pattern, **x**. As the model output is a linear combination of the neuron activation functions output Equations (1), (3) can be given as,

(4) $$\Phi ({\text{X}}^{t},\text{w},\text{C},\text{σ})=\frac{1}{2}{\left\|\text{y}-\phi ({\text{X}}^{t},\text{C},\text{σ})\text{w}\right\|}^{2}$$

where omitting the dependence of ***φ*** on **C** and ***σ***,

$$\phi ({\text{X}}^{t},\text{C},\text{σ})={\left[\varphi (x(1))\varphi (x(2))\cdots \varphi (x(N))\right]}^{T}$$

By computing the global optimum value (**w**^∗^) of the linear parameters **w**, with respect to the nonlinear parameters **C** and ***σ***, as a least-squares solution,

(5) $${\text{w}}^{*}=\phi^{+}({\text{X}}^{t},\text{C},\text{σ})\text{y}$$

where “+” denotes a pseudo-inverse operation, and replacing Equation (5) in Equation (4), the training criterion to determine the nonlinear parameters **C** and ***σ*** is:

(6) $$\Psi ({\text{x}}^{t},\text{C},\text{σ})=\frac{1}{2}{\left\|y-\phi ({\text{X}}^{t},\text{C},\text{σ})\phi^{+}({\text{X}}^{t},\text{C},\text{σ})y\right\|}^{2}$$

The initial values for the neuron centre positions are randomly selected from the training data, and the spreads of the neuron activation functions are initialised using a simple and straightforward rule in [38]. The training procedure progresses iteratively using the LM algorithm minimising criterion Equation (6), until a termination criterion is satisfied. Once the models are trained, their parameters may be adapted on-line [39], for instance, if the process is time-varying. For complete details about the training algorithm, the training criterion and termination criteria, the reading of [18,19,29,35] is recommended.

The design of an RBF ANN is only complete once the model input-output structure, *i.e.*, its number of neurons and input terms, is selected. In classification problems a limited number of input features must be selected from a larger set. In non-linear regression problems, for instance when designing Non-linear Auto Regressive (NAR) or NAR with Exogenous inputs (NARX) predictive models, the input variables and their delays must be selected. These are multi-criteria combinatorial optimisation problems where exhaustive search easily becomes computationally prohibitive. In order to address this part of the model design, the MOGA is employed to evolve a *preferable* set of models whose number of neurons and selected input terms optimise pre-specified goals and objectives. The approach is described in the following subsection.

### 2.2. RBF Topology and Inputs Selection

The MOGA, as well as other MOEAs, are one class of EAs that use a set of procedures and operators in order to perform a population based search for the Pareto set of solutions of a given multiobjective problem. The solution candidates are called *individuals* and their set is referred to as the *population*. One run of a MOGA starts with an initial population of individuals, the initial *generation*, which are then evaluated and manipulated to compute the population of the next generation. The manipulation consists in using appropriate operators to perform analogue operations to mating selection, chromosome recombination and mutation, on the individuals. Hopefully, after a sufficient number of generations the population has evolved, thereby achieving a satisfactory approximation to the Pareto front.

Each individual in the MOGA population must be specified by a representation, the *chromosome*, which in this case encodes the topology of a RBF ANN. This may be specified by the number of neurons, *n*, and by the indices to a lookup table of possible inputs. Therefore, the chromosome is a string of integers, the first representing the number of neurons and the remaining representing the subset of inputs selected from a given set of possibilities. When the number of inputs is variable, as in the RBF models to be described, this representation is known as a *variable length chromosome*.

Once the population has been evaluated and each individual is assigned a fitness value, the mating procedure initiates the evolutionary process. It is implemented by a sampling procedure where the probability of one individual being sampled is related to that individual's fitness evaluation. The outcome is that the fittest individuals have a higher probability of breeding as opposed to the worse individuals that are unlikely to influence the new generation.

With a given probability, *i.e.*, the *crossover probability*, for every pair mated the recombination operator produces two offspring by exchanging part of their chromosomes. First the chromosomes are reordered, then, beyond the *crossover point* in the string, the parts are exchanged. The crossover operation implemented is known as *full identity preserving crossover*[40,41], as it guarantees offspring with no duplicate terms.

Mutation is then applied to the new population, independently in two parts of the chromosome. The number of neurons of the RBF ANN is mutated, with a given probability, by adding or subtracting one neuron to the existing quantity. Boundary conditions, *n_m_* ≤ *n* ≤ *n_M_*, are checked, where *n_m_* and *n_M_* are the minimum and maximum allowed number of neurons, respectively. The input terms are mutated, with a specified probability, by one of three operations: replacement, addition or deletion. First, each term is tested and is either deleted or replaced by another term not in the chromosome. Deletion only occurs if the chromosome has more terms than the minimum specified, *d_m_*. After this, if the chromosome is not full (length *d_M_* + 1), one term may be added by selecting it from the set of those outside the chromosome.

After completing the operations just described, the MOGA proceeds to the evaluation stage and the cycle repeats itself for the new population of individuals.

**Figure 2.**
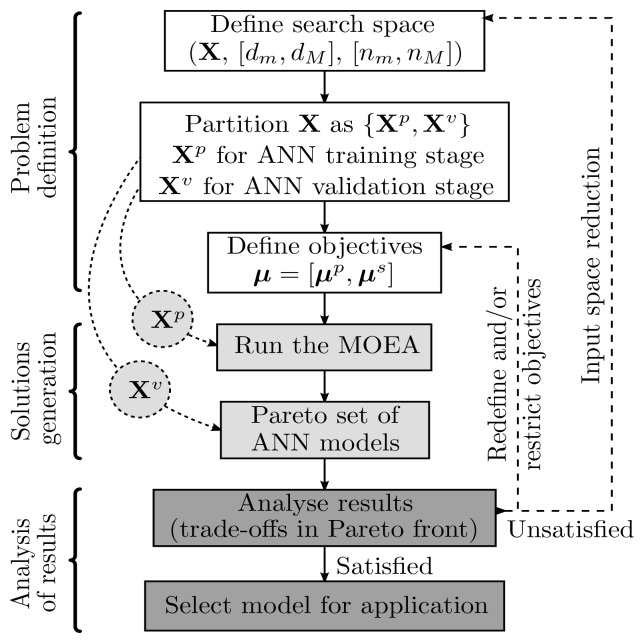
Model design cycle.

Globally, the ANN design procedure can be viewed as the sequence of actions undertaken by the model designer, which should be repeated until pre-specified design goals are achieved. These actions can be grouped into three major categories: problem definition, solution(s) generation and analysis of results. In the context of the identification framework used, the procedure is executed as depicted in Figure 2. **X** represents the data available for the model identification. It is usually an *N* × *d* matrix with *N* points or patterns and *d* features. These are, in the case of non-linear regression problems, delayed values of the input variables. **X** is partitioned into **X***^p^* and **X***^v^*, the first data set for RBF ANN parameter estimation using the methods described in Section 2.1, the second to undertake a final model evaluation after the execution of the MOGA. It should be noted that **X***^p^* may be further decomposed into **X***^t^* and **X***^g^* in case some form of generalisation testing is used either to stop the training procedure using early stopping or to include generalisation ability measures in the MOGA objectives. These, denoted by *μ* in Figure 2, are also subdivided into ***μ****^p^* and ***μ****^s^*, to emphasise the distinction between objectives related to the ANN training stage (***μ****^p^*) and to the ANN fitness for the specific application (***μ****^s^*). Typical elements of ***μ****^p^* are the ANN Root Mean Square (RMS) of the training and generalisation errors and some form of ANN complexity measure.

### 2.3. Model Evaluation

In the model identification experiments that will be presented, two or three objectives were used in the MOGA to assess the models fitness. To ease their description, let the RMS function operating over the *ith* column of its argument matrix be denoted as *ρ* (·, *i*). Also, consider the following error matrix computed on the basis of the model prediction error taken from the multi-step model simulation over a prediction horizon *ph*, using a data set **X** with *p* input points:

$$\text{E}(\text{X},\text{ph})=\left(\begin{array}{cccc} e_{1,1} & e_{1,2} & \cdots & e_{1,\text{ph}}\\ e_{2,1} & e_{2,2} & \cdots & e_{2,\text{ph}}\\ \vdots & \vdots & \ddots & \vdots \\ e_{p-\text{ph},1} & e_{p-\text{ph},2} & \cdots & e_{p-\text{ph},\text{ph}} \end{array}\right)$$

where *e_i,j_* is the model prediction error taken from instant *i* of **X** (*ith* row of **X**), at step *j* within the prediction horizon, and **E**(**X**, 1) is the one-step-ahead model prediction error vector for the input data set **X**. When *ph* > 1, starting at *j* = 2, the one-step-ahead predictions are used to alter **X** in accordance with the model inputs definition, so that the two-steps-ahead prediction is obtained. By repeating the process up to *ph*-steps-ahead, **E**(**X**, *ph*) is formed.

By means of these definitions, the objectives may be formulated as:

(7) $${ɛ}^{t}=\rho (\text{E}({\text{X}}^{t},1),1)$$

(8) $${ɛ}^{g}=\rho (\text{E}({\text{X}}^{g},1),1)$$

(9) $${ɛ}^{s}=\sum_{i=1}^{\text{ph}}\rho (\text{E}({\text{X}}^{s},\text{ph}),i)$$

The first two objectives are simply the RMS of the ANN training and generalisation errors, considering the **X***^t^* and **X***^g^* data sets, respectively. These are used in the identification of models for cloudiness estimation as well as in the identification of the predictive models. The third objective, used only in the identification of predictive models, is a long term prediction performance measure which reflects the model's predictive ability over the complete prediction horizon. It corresponds to the sum of the RMS of the columns of **E**(**X***^s^*, *ph*).

## 3. Cloudiness Estimation and Prediction

The existing research in cloudiness differs in the purpose, on how data is acquired and on how it is processed to extract useful clouds information. The most commonly used technologies for acquisition are satellites, ground-based all-sky imaging systems, ceilometers, radars, lidars, and spectroradiometers. In the detection of clouds, satellites tend to be used more commonly, as shown by several studies [42–45]. Ground-to-sky imaging systems tend to be a cheap alternative that renders images with greater resolution at increased acquisition frequency and also provides more focused results [46–51]. In addition, they allow a view of low level clouds, which are often hidden by high clouds when observed from satellites.

By means of images acquired with a ground-to-sky imaging system, an RBF ANN was identified with the purpose of estimating a threshold on a given pixel intensity scale that segments the input image pixels into the cloud or clear sky classes [23]. This ANN was used afterwards to build cloudiness time-series, which in turn were employed in the identification of an auto-regressive cloudiness predictive ANN model [24]. As these ANNs are used in the intelligent sensor that was developed, their identification will be described in the following subsections and, in the case of the image segmentation ANN, its comparison with two well known image segmentation algorithms will be presented.

### 3.1. Cloudiness Estimation

#### 3.1.1. Ground-Based All-Sky Images

**Figure 3.**
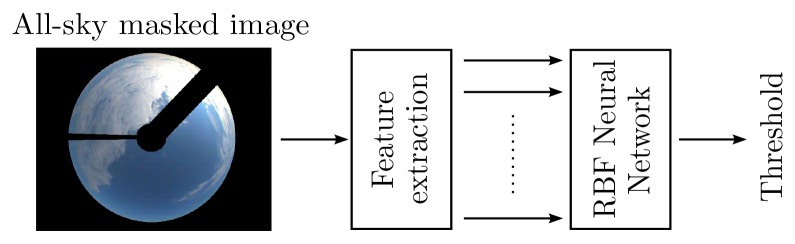
Neural network thresholder approach.

In total, 410 all-sky images (704 × 576, *Red-Green-Blue* (RGB), 8 bit/channel) were acquired using a *Total Sky Imager (TSI) 440A* manufactured by Yankee Environmental Systems, Inc. (Turners Falls, MA, USA), located on top of one building (37°02′*N*, 07°57*W*) in the University of Algarve, Faro, Portugal. Care has been taken in order to cover a wide range of cloud cover situations as well as their distribution over daytime. Details may be found in [23]. By using location coordinates and the time of acquisition, a pixel mask was computed to identify the visible sky pixels for further processing (see Figure 3 for an example). The masking operation filters all pixels that are not clouds or clear sky. Afterwards, one researcher made an additional mask including all the cloud pixels according to his judgment. By using these masks the reference percent cloud cover was computed using the formula,

(10) $$C=\frac{N_{c}}{N_{s}+N_{c}}\times 100$$

where *N_s_* and *N_c_* are the number of pixels masked as clear sky (class **S**) and cloud (class **C**), respectively. Additionally, for each pixel intensity scale studied, an exhaustive search was conducted to find the reference threshold value, *t_o_*, minimising the cloud cover estimation error resulting from the threshold operation.

#### 3.1.2. Thresholding Methods Considered

The approach, common to all methods, consists in finding a threshold value, *t̂*, on a given pixel intensity scale, which segments the image *I* pixels with coordinates (*x*, *y*) and intensity *i_xy_* into one of the classes, **S** and **C**. In this sense these are sets defined as,

(11) $$\text{S}=\{(x,y)\in I:i_{\text{xy}}\leq \hat{t}\}$$

(12) $$\text{C}=\{(x,y)\in I:i_{\text{xy}}>\hat{t}\}$$

to which *N_s_* and *N_c_* in Equation (10) are the respective set cardinalities. The evaluation of the thresholding methods relies on the absolute error between the reference cloud fraction attributed to the images and that estimated by the threshold:

(13) $${ɛ}^{c}=\left|C-{\hat{C}}_{t}\right|$$

Several pixel intensity scales were considered for the thresholding operation. From the results in [23] one was selected, denoted *hsvR*, as it consistently provided increased cloud cover estimation accuracy for all the methods tested. This pixel intensity is obtained by:

1. converting the original image to the Hue-Saturation-Value (HSV) colour model;
2. setting the V channel to 1 (the maximum) in all pixels;
3. converting this image back to the RGB mode;
4. selecting the red channel for the thresholding operation.

Setting an equal value on the V channel has an equalisation effect on the pixels luminosity. The maximum was chosen because on the HSV model the colours become more distinguishable. The net effect on the converted RGB image is that clear sky and cloud pixels have improved contrast between them in the red channel. Four different threshold methods were considered:

##### Fix threshold

A histogram analysis was made to identify the best single threshold value that could be applied to all the images. Global (over all images) pixel intensity probability mass functions (PMF) were separately computed for each of the classes, **S** and **C**, and a search was conducted in the vicinity around the intersection point of the PMFs in order to find the fixed threshold that minimises the average (over all images) value of Equation (13). This was found to be *t* = 158 on the *hsvR* pixel intensity scale.

##### RCT method

This method is a histogram-iteration form, presented in [52], of an iterative thresholding algorithm [53] that we denote by RCT in the following. A brief description of the method may be found in [23]. For a more in-depth view the reader should consult [52–55].

##### Otsu's method

The principle behind the method proposed by [56] is very simple: an exhaustive search is conducted on the pixel intensity scale for the threshold that maximises the inter-class variance. Again a brief overview may be found in [23], whereas for more detailed descriptions [55,56] may be consulted.

##### Neural network method

Considering that on average the RCT and Otsu methods could not overcome the performance of the single threshold approach, an attempt was made to identify an RBF ANN image thresholder by using the model identification framework presented above. As illustrated in Figure 3, the output of the ANN is the threshold to be used in an image, and the inputs are a certain set of features extracted from that image.

The set of 410 images was broken into three sub-sets: training set (**X***^t^*, 290 images), generalisation testing set (**X***^g^*, 60 images), and the validation set (**X***^v^*, 60 images). From all the images and from transformations of them, a total of 69 input features were extracted. The complete list of features is specified in [23]. It includes histogram properties and the first three statistical moments of the various image intensity channels considered.

The stopping criteria used to terminate the training process was the *early stopping* method by means of **X***^g^*. As the RBF ANN parameters are initialised randomly, 25 training trials were executed and the average values of the objectives were employed as fitness measures. This procedure decreases the chance of one potentially good model being poorly evaluated due to the chance of being badly initialised, and increases the likelihood of better models achieving better fitness when compared to worse models.

The RBF ANN structure was selected by the MOGA as described previously. Table 1 shows the parametrisation of the model identification experiment. The search space was defined by allowing from 2 to 36 input features and by letting the number of neurons vary between 2 and 24. This results in a search space with approximately $\left[\sum_{k=2}^{36}\left(\begin{array}{c} 69\\ k \end{array}\right)\right]\times 23\approx 9.3\times 10^{21}$ possibilities. Two objectives were set up for minimisation: *ε^t^* and *ε^g^*. The last four rows of the table show the parameters related to the MOGA and its genetic operators.

**Figure 4.**
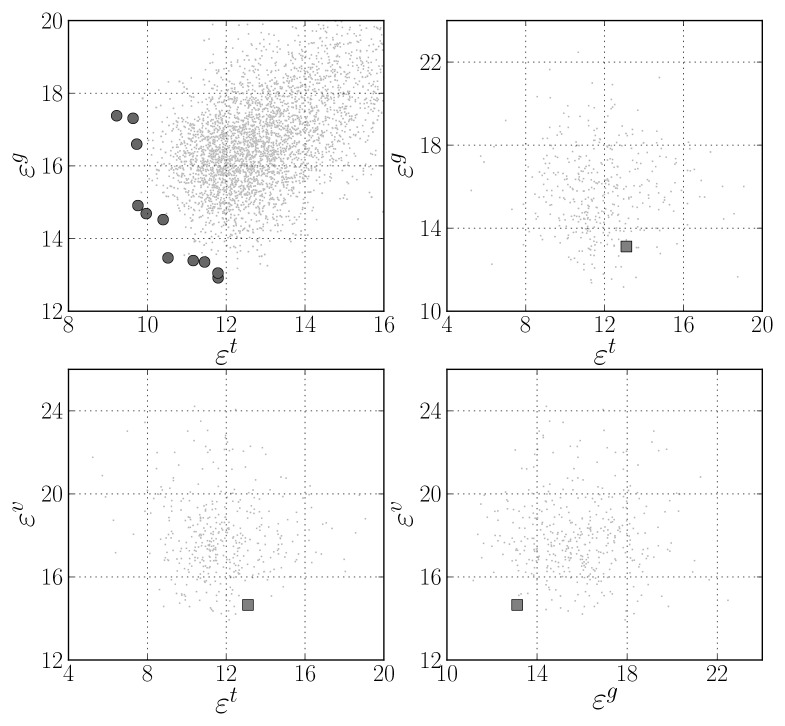
ANN thresholding model selection. Top-left: objective values after MOGA execution (dark circles denote Pareto set). Remaining plots: Results after executing 50 training trials for each of the models in the Pareto set (dark square shows select model instance).

After 50 generations a set of 11 models were evolved, which are preferable on the specified objective space. These are highlighted by dark circles in the top-left plot of Figure 4, where a detail of the resulting objective space is shown. Regarding the number of neurons of the eleven selected models, four had from 12 to 14 and the remaining seven had 22 or 23 neurons. The number of input features varied from 29 to 36. The most frequent input features present in all models were the most frequent intensity of the RGB image R channel, the skewness of the saturation channel in the HSV image, and the standard deviation of the saturation and lightness channels of the Hue-Saturation-Lightness image.

Considering that the objectives are average values of several training trials, for each of the preferable ANN models, 50 additional training trials were executed in order to select one final ANN for the thresholding application. This choice was made by taking into account the actual objective values attained on each of the trials and also the RMS error obtained on the validation data set, *ε^v^* = *ρ*(**E**(**X***^v^*, 1), 1). The results are shown in the top-right and in the bottom plots of Figure 4. Those marked with a dark square were obtained by the RBF ANN that was selected after analysing all the numbers. It presented the most favourable balance in the objectives, achieving the RMS error values of 13.10, 13.12 and 14.65 respectively on **X***^t^*, **X***^g^* and **X***^v^*. It is a network with 30 input features and 22 neurons.

Figure 4 provides results only on the image threshold estimation accuracy. Table 2 shows how these results translate into cloudiness estimation accuracy by means of Equation (13) and provides the results achieved by the other methods considered. The first three rows present the results obtained considering the training and testing data sets together (involved in the MOGA RBF optimisation), the validation data set alone, and the three data sets altogether. The outcome is an accuracy improvement of approximately 50% when compared to the best results obtained by the remaining methods.

### 3.2. Cloudiness Time-Series Prediction

**Figure 5.**
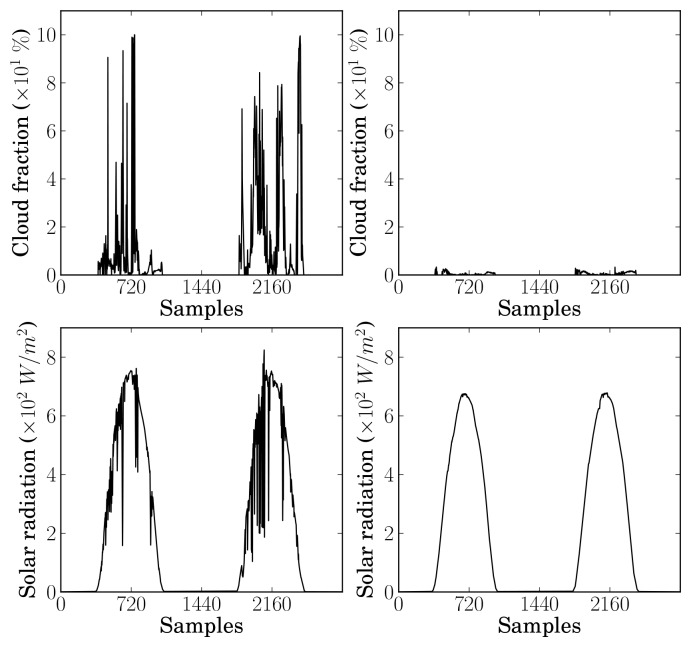
Two examples of two consecutive days of estimated cloud fraction and measured solar radiation.

Using the selected RBF ANN image thresholder, the cloudiness time-series were estimated by feeding the ANN with the features extracted from consecutively acquired images (1 min sampling interval), and applying (Equations 10–13) for each image and the corresponding estimated threshold. As an example, Figure 5 illustrates (top plots) the resulting cloudiness time-series for two groups of two consecutive days. The measured global solar radiation is also shown (bottom plots) so that the relation to cloudiness may be visually inspected.

These time-series were afterwards employed in the identification of RBF ANN cloudiness predictive models. As these are meant to be employed in a global solar radiation prediction scheme operating on the basis of a 5 min sampling interval, the cloudiness time-series were averaged over 5 min consecutive periods, thus generating smoothed time-series synchronised to the solar radiation data.

The data from the generated time-series was split into four data sets: the training data set (**X***^t^*), the generalisation data set (**X***^g^*), the validation data set (**X***^v^*) and a simulation data set (**X***^s^*). **X***^s^* is used to compute *ε^s^* (remember Equation (9) in Section 2.3), in order to evaluate the models in long-term prediction.

The identification experiment of RBF ANN cloudiness predictive models followed the same approach outlined for the ANN image thresholder and was parametrised as presented in Table 3, where the MOGA specific parameters are the same as presented in Table 1 and hence not repeated.

In this case, the models were identified as NAR one-step-ahead predictive models that may be expressed by the relation

(14) $${\hat{y}}_{k+1}=f(y_{k},y_{k-1},\ldots ,y_{k-i},\ldots ,y_{k-d})$$

where *f* is a non-linear function, in this case an RBF ANN. In order to complete a forecast up to a specific prediction horizon *ph*, the model is iterated in a multi-step fashion to generate predictions from instants *k* + 1 to *k* + *ph*:

$$\begin{array}{lll} {\hat{y}}_{k+2} & = & f({\hat{y}}_{k+2},y_{k},\ldots ,y_{k-d+1})\\ & \vdots & \\ {\hat{y}}_{k+\text{ph}} & = & f({\hat{y}}_{k+\text{ph}-1},{\hat{y}}_{k+\text{ph}-2},\ldots ,y_{k-d+\text{ph}-1}) \end{array}$$

For such a model, the selection of inputs corresponds to the selection of the delays that should be considered from the set {0, 1, …, *d*}. The set of delays allowed at the model input were defined from a 24 h range that, at 5 min sample interval, would correspond to 288 possibilities. In order to decrease this number, the density of delays within the 24 h was varied: from the latest 8 h the delays considered were taken at 15 min intervals, from the previous 8 h they were taken at 10 min intervals, and finally, from the first 8 h all the delays were considered. Through this way, the number of possible inputs was decreased from 288 to 176. By considering this and the number of neurons allowed, the number of possible models in the search space is $\left[\sum_{k=2}^{24}\left(\begin{array}{c} 176\\ k \end{array}\right)\right]\times 17\approx 4.9\times 10^{30}$. In this expression, the limits on *k* correspond to the range allowed for the number of inputs, therefore the sum gives the number of possibilities of taking those numbers of inputs from the 176 available. The grand total is obtained by multiplying the sum by the number of possibilities for the number of neurons, in this case 17 (from 4 to 20). In addition to the model training and generalisation ability objectives in ***μ****^p^*, in this experiment an application specific objective *ε^s^* was also included in order to obtain the best predictive ability within the 48 steps horizon. The training RMS output error was set up as a restriction of 8%. As the ANN model parameters are randomly initialised, 10 training trials were executed and the average values of *ε^t^* and *ε^g^* were used. In order to decrease the computational load, *ε^s^* was only computed for the model whose pair (*ε^t^*, *ε^g^*) was closer (in the Euclidean sense) to the averages over the 10 trials, $\left(\bar{\varepsilon^{t}},\bar{\varepsilon^{g}}\right)$.

From the MOGA execution 5 models were obtained in the preferable set, after 116 generations.

**Figure 6.**
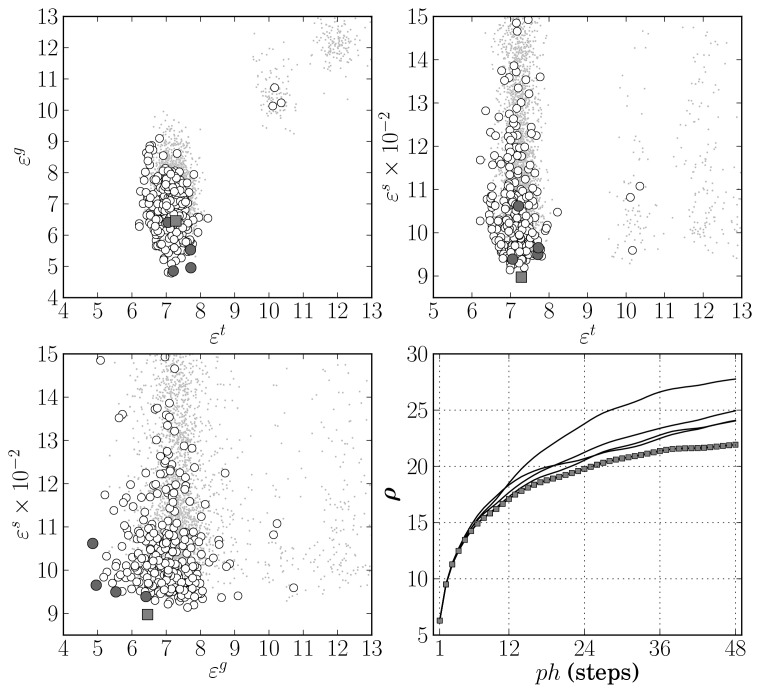
Relation between the objectives of the cloudiness predictive model identification experiment. White circles mark non-dominated solutions, dark circles denote the preferable set of models, and the grey square shows the model structure that was selected. The lower-right plot shows the evolution of *ρ*(**E**(**X***^s^*, *ph*), *i*) with *i* within the prediction horizon *ph*. The curve with grey square markers was obtained by the model marked with a grey square in the remaining plots.

Figure 6 illustrates the results obtained in the space of objectives specified to the MOGA. The three scatter plots show the non-dominated model results using white circles, and the results of the preferable set of models using dark circles and one grey square. The lower-right plot presents the evolution of *ρ*(**E**(**X***^s^*, *ph*), *i*) along the prediction horizon, denoting the results obtained by the model marked with a grey square on the remaining plots also with grey square markers. All the models had 5 or 6 neurons and from 12 to 18 input terms.

**Figure 7.**
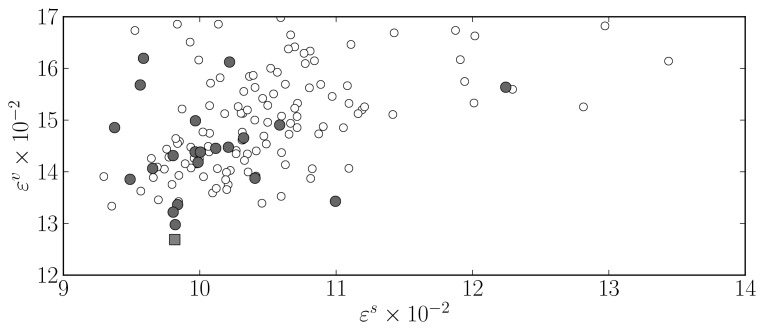
Long term prediction performance on simulation dataset, *ε^s^*, *versus* validation data set, *ε^υ^* (not used in MOGA), for the 5 preferable models. Dark circles correspond to results achieved by the model structure denoted with a grey square in Figure 6. The dark square denotes the selected model instance.

These 5 models were evaluated on **X***^υ^*, by computing ${ɛ}^{\upsilon }=\sum_{i=1}^{\text{ph}}\rho (E(X^{\upsilon },\text{ph}),i)$, in order to avoid any bias towards **X***^s^* that may have occurred during the MOGA model optimisation. As during the MOGA execution only one out of 10 instances were used to compute *ε^s^*, 50 additional training trials were executed for each of the preferable models and the final selection was made on the basis of *ε^s^* and *ε^υ^*. The results are presented in Figure 7.

The selected model is a network with 6 neurons and employing 13 input terms. Coincidentally, this model structure corresponds to the best one in the preferable set in terms of the long term prediction objective, *ε^s^*, and is shown by a dark square in Figure 7. It has been employed to improve the identification of global solar radiation models as will be described in the following section.

## 4. Solar Radiation and Air Temperature Prediction

As with the cloudiness time-series predictive model, the objective is to predict the global solar radiation and air temperature profiles over a prediction horizon of 4 h (48 steps at 5 min sampling interval). Most related work found in literature is focused on estimating the daily irradiance by means of various methodologies such as stochastic models [57], empirical methods [58], thin-plate splines [59], or satellite images [60]. ANNs have also been applied for that purpose [61,62] and studies have shown that they outperform other physical and empirical approaches [63]. In order to dynamically forecast the global solar radiation evolution within relatively short-term horizons, stochastic models have been used to obtain the profile over a day [57], and ANNs have been employed to predict the profile over a few hours horizon at rates from 1 to 15 min [20–22]. The models that have been implemented in the prototype device were identified [64] by improving from these previous ANN approaches.

Regarding the air temperature prediction, extensive work may be found on the prediction of daily and seasonal temperature values. The dynamical forecast within shorter horizons, from minutes to a few hours, has been less reported. In this case, ANNs seem to be the preferred method for horizons of a dozen hours [65] where support vector regression was also considered, but also to generate forecasts from several minutes to a few hours [26,30]. In order to improve existing results, RBF ANN models have been identified [64] and implemented in the prototype device.

### 4.1. Solar Radiation Predictive Models

The ANN models were identified using the techniques already outlined, in a similar fashion as described for the cloudiness predictive model, by applying the MOGA to identify the ANN's input-output structure. Two approaches have been used, the first consisting in an NAR approach and the second consisting in an NARX approach using cloudiness as an exogenous input. Cloudiness affects significantly the diffuse solar radiation, which justifies its inclusion at the inputs of solar radiation models. For the NAR case, the allowed input delays were specified in the same way as for the cloudiness time-series predictive model, in order to capture the daily pattern. For the NARX case these input delays were also considered, and in addition, all the delays from the exogenous variable within the 4 h prior to the prediction instant were also allowed in order to capture the dynamics imposed by diffuse solar radiation. This results in 176 and 224 possible input terms for the NAR and NARX approaches, respectively. The MOGA was parametrised as presented in Table 4, where the MOGA specific parameters are omitted since they are the same as presented in Table 1. Given these model structure parameters, the number of possible models in the search space are, for the NAR and NARX approaches, $\left[\sum_{k=2}^{32}\left(\begin{array}{c} 176\\ k \end{array}\right)\right]\times 15\approx 2.6\times 10^{36}$ and $\left[\sum_{k=2}^{32}\left(\begin{array}{c} 224\\ k \end{array}\right)\right]\times 17\approx 1.1\times 10^{40}$, respectively. The objective space was also specified as for the cloudiness time-series predictive model, including the approach to compute *ε^s^* only for one model out of 10 training trials.

After the execution of nearly 100 generations, eleven models resulted in the preferable sets of both the NAR and NARX approaches. For the first approach, the models had from 13 to 25 inputs, where five employed 9 neurons and the remaining employed more than 10. For the second approach, the number of inputs varied from 15 to 28, where one model had 6 neurons and the remaining ten had 7.

**Figure 8.**
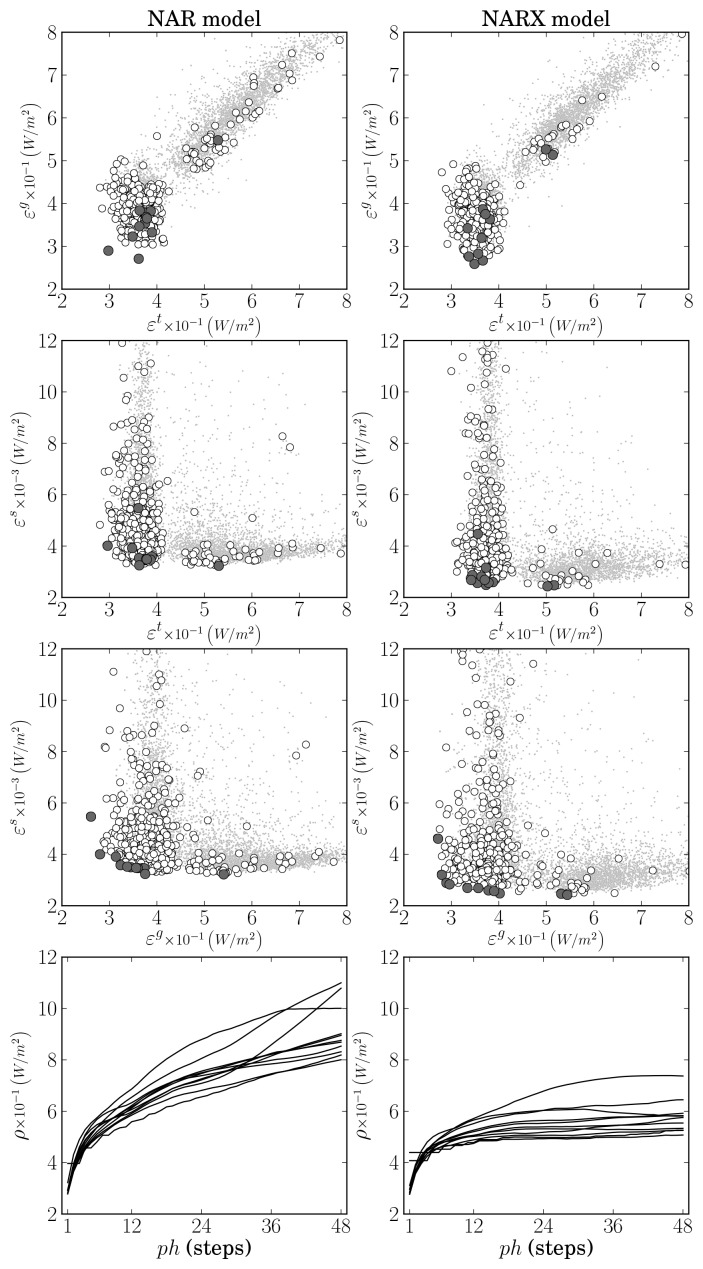
Relation between the objectives achieved by NAR and NARX global solar radiation predictive models. Left and right plots correspond to the NAR and NARX modelling approaches, respectively. White circles mark non-dominated solutions, darker circles denote the preferable set of models, and the grey small dots mark remaining solutions. The two plots at the bottom show the evolution of *ρ*(**E**(**X***^s^*, *ph*), *i*) with *i* within the prediction horizon *ph*.

The results obtained in the space of objectives are illustrated in Figure 8. The column of plots on the left are related to the results achieved by the NAR approach, whereas the column on the right relates to the NARX approach.

By looking at the scatter plots, it may be concluded that generalisation has been slightly improved by the introduction of cloudiness data at the input of the solar radiation model. Although the training results (*ε^t^*) are similar, a decrease in generalisation error (*ε^g^*) is observed. A probable (and more important) consequence of this is the decrease in long-term prediction ability achieved by the NARX approach. The results shown in the plots at the bottom of Figure 8 exhibit a decrease around 35% in the error at the end of the prediction horizon. However, it should be noted that the minimum error approach was employed when computing *ε^s^*, *i.e.*, whenever future values of the cloudiness were necessary to compute one prediction within the considered horizon, the measured values were employed. As a result, in a practical application where the cloudiness predictive model would first be used to obtain the necessary predictions, the results would necessarily be worse than the limiting case obtained. Still, there is a 35% margin for the NARX approach to be preferred over the NAR one.

### 4.2. Air Temperature Predictive Models

For the air temperature models, only the NARX approach was followed with solar radiation as the exogenous variable, as it was established previously by [66] that it is preferable to the NAR model for long-term prediction. Air temperature is affected by direct sunlight, whose dynamics are well captured by solar radiation. The experimental methodology was similar in all aspects to the NARX solar radiation predictive model identification experiment described in the previous subsection. The exceptions are (1) the number of delays of the solar radiation that were considered as possible inputs, specifically all within the 6 h prior to the prediction instant, and (2) the specific MOGA identification experiment parameters that are shown in Table 5. For this modelling experiment, the resulting number of possible models in the search space is $\left[\sum_{k=2}^{32}\left(\begin{array}{c} 248\\ k \end{array}\right)\right]\times 19\approx 6.4\times 10^{40}$.

**Figure 9.**
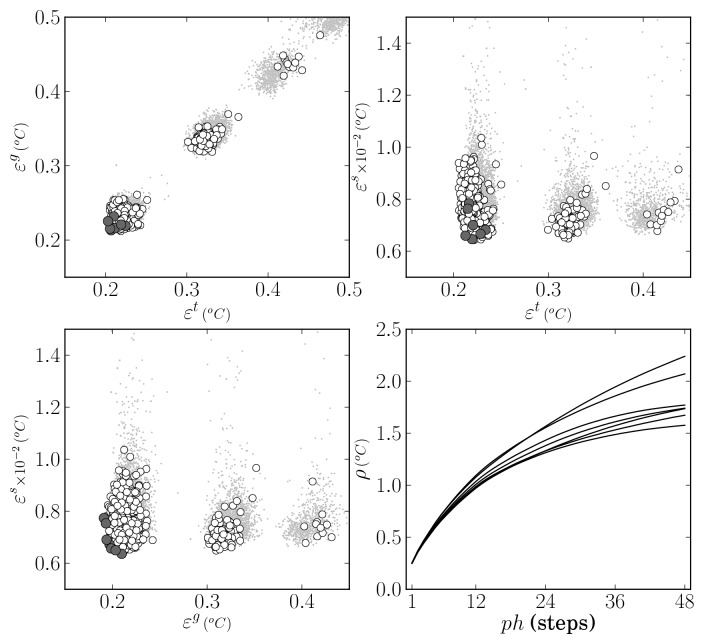
Relation between the objectives of the air temperature predictive model identification experiment. White circles mark non-dominated solutions, dark circles denote the preferable set of models. The lower-right plot shows the evolution of *ρ*(**E**(**X***^s^*, *ph*), *i*) with *i* within the prediction horizon *ph*.

The MOGA was executed for 200 generations, producing seven models in the preferable set. These had from 15 to 25 inputs, where six had 4 neurons and the remaining one had 5. Regarding the identification experiment objectives, the results are shown in Figure 9.

It may be seen that the preferable set of models achieves excellent generalisation and predictive performance. Within the prediction horizon, the RMS error is about 1.25 °*C* after 2 h and 1.5 °*C* after 4 h for the best model. Again, it must be noted that these results were obtained by using the minimum error prediction approach, as explained before, and therefore will suffer from some deterioration.

### 4.3. Implementation Alternatives

By taking into account the models that were identified and described in previous sections, two options are available for the implementation of predictive models in the intelligent sensor. One consists in using two cascades of two models and the other consists in using a cascade of four models:

#### Option 1

One cascade is composed of the cloudiness estimation model that feeds the NAR cloudiness time-series predictive model, and the second cascade connects the NAR solar radiation predictive model to the NARX temperature predictive model.

#### Option 2

The cascade of four models consists of the first cascade in option 1 feeding the NARX solar radiation model, which in turn connects to the NARX temperature model.

As a final note about the models, it should be mentioned that the same selection strategy used to select the cloudiness time-series predictive model was employed to choose the solar radiation and temperature models that were implemented in the prototype device.

## 5. Prototype Device

The development of the prototype intelligent sensor was planned by having the *TSI* system as a reference for the design and for future comparison of results. The *TSI* structure has two parts, a chrome-plated steel mirror on which the whole sky is reflected on, and an arm that holds an *Axis*200+ IP camera that captures the reflection of the sky in the mirror. The acquisition of the images is done by accessing the IP camera web server. This device requires remote data storage over TCP/IP connection and manual calibration on operation site, weighs approximately 23 *kg*, and does not include any other sensor.

A prototype was developed to take direct (no reflecting mirror) all-sky photographs, including solar radiation (Kipp & Zonen SP Lite2) and air temperature (LM35 DZ) sensors, a GPS device to ease and automate setup procedures, and a small computer to provide storage, model execution capabilities and connectivity. The aim was to achieve increased functionality, improved portability, therefore targeting a broader range of applications. The sky photographs and the sensor measurements are fed to the set of neural networks as described above, in order to provide the forecasts as illustrated in Figure 1.

A video camera (Genie CCTV camera) was used with a fish-eye lens in order to capture the largest possible area of the sky. The common vignetting effect introduced by fish-eye lenses was decreased because part of the images periphery is masked in order to remove visible buildings. With compactness and lightness in mind, the prototype was designed to aim the video camera directly at the zenith, therefore removing the need for a heavy and expensive reflecting mirror and a structure to fix the camera above the mirror, and consequently requiring less space. Without any protection from the sun, even with an auto-iris function, the camera could not avoid significant glow and lens flare. To overcome this, a shadow band was implemented by means of a PVC stripe positioned above the lens. The position of the stripe is controlled by an unipolar stepper motor (NMB PM55L-048-HP69) and determined using a sun path algorithm [67,68], which computes the azimuth and elevation angles as well as sunrise and sunset times as a function of the time, date, latitude and longitude data acquired by the GPS device (Globalsat EM406-A).

A simple micro-controller (Arduino Duemilanove) was used in order to acquire measurements from the sensors and GPS, to control the shadow band position and to interface these devices to the computer (Ebox 4300). The video camera was interfaced to the computer using an USB video capture card. The computer has a Via Eden ULV 500 MHz CPU and runs a GNU/Linux operating system. All the models and system routines were implemented using the C and Python languages.

**Figure 10.**
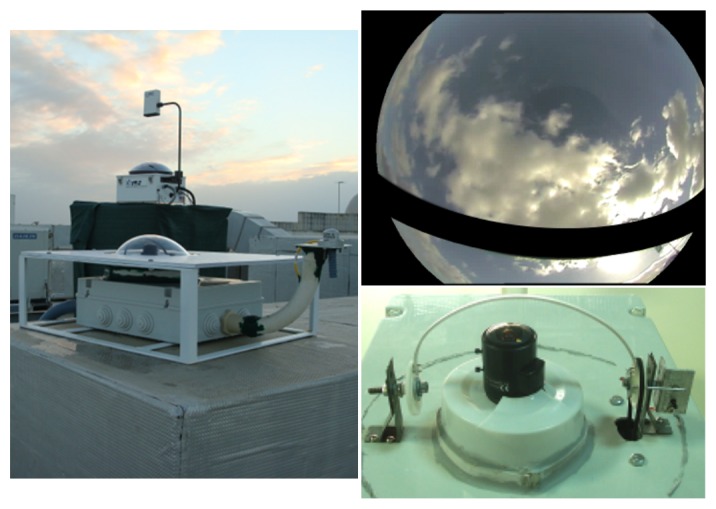
**Left:** prototype system in the front with *TSI* system in the background. Bottom: Shadow band above lens. **Top:** Example of acquired image after masking.

Figure 10 presents pictures from the prototype system in operation, a detail from the shadow band and an image acquired by the device (after masking operation). The system was assembled within an enclosure suitable for outside operation, which protects electric components from rain, wind and direct sunlight exposure.

## 6. Results and Discussion

Testing and validation was performed firstly by comparing the results obtained by the prototype to those obtained on a laboratory computer, using the same input data. The aim was to validate the implementation of the models in the prototype. The numerical errors obtained were due to the different floating point architecture of the systems, in the order of 10−^14^, confirming the compilation and implementation of models in the prototype.

Another test consisted in the comparison of results obtained by the prototype to those obtained by a reference system. For the case of cloudiness, the *TSI* system was used as a reference. For solar radiation and air temperature, the reference was given by a Delta-T weather station installed a few meters away from the *TSI* and the prototype. This station has a BF3 sensor for solar radiation and a RHT2 sensor for air temperature. These tests have the purpose of validating the sensors and data acquisition hardware.

The last validation performed consisted in the comparison of the predictions obtained by means of images acquired by the prototype with those obtained using images acquired by the *TSI*. The data used in the validation was acquired between 28 January 2011 and 30 January 2011. The first day was used to initialize both NAR and NARX models, while the two remaining days where used for the comparison of predictions. The following subsections present the results of the tests for cloudiness, solar radiation and air temperature.

### 6.1. Cloudiness

**Figure 11.**
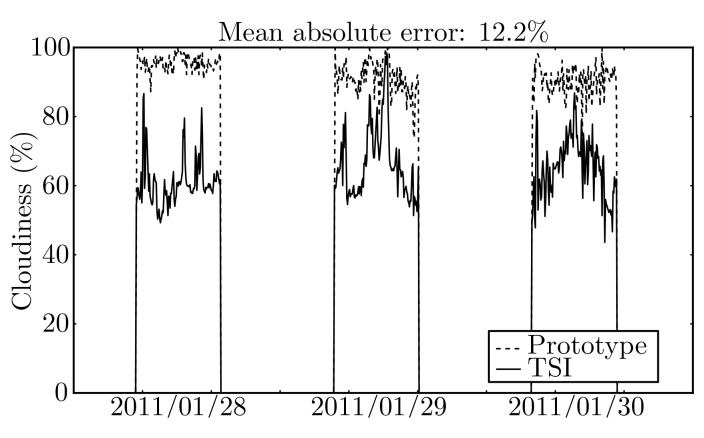
Estimated cloudiness using images from the prototype and from *TSI*.

The cloudiness estimated by means of prototype images and that estimated from images acquired using the *TSI* are shown in Figure 11.

**Figure 12.**
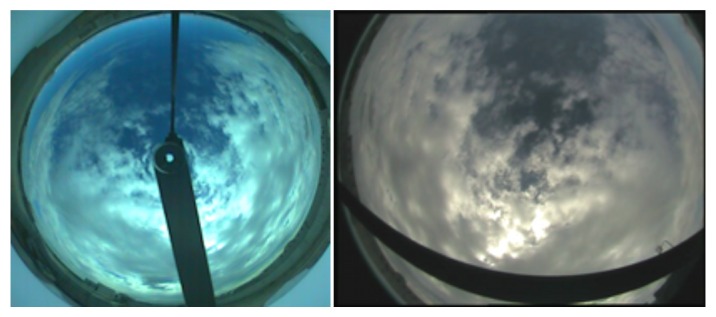
Images acquired by the prototype (**right**) and the *TSI* (**left**) at the same time instant. The different tonality is evident.

The mean absolute error between the two systems is 12.2% with a variance of 15.4%. The difference observed between the two systems was not unexpected and is due to the fact that the employed ANN was trained to provide a threshold using images from the *TSI*. When the ANN is applied to images with a different tonality, as those acquired by the prototype, it induces an error in the cloudiness estimation. Figure 12 provides an example that clearly shows the difference of the images.

**Figure 13.**
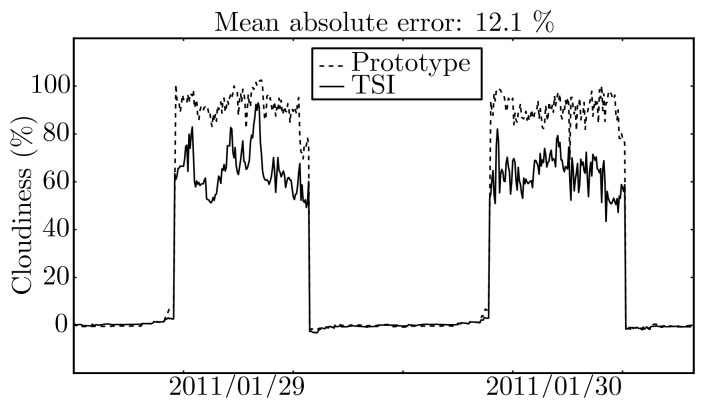
One-step-ahead prediction of cloudiness using images from the prototype and from the *TSI*.

The last comparison regarding cloudiness is illustrated in Figure 13, which shows the one-step-ahead (5 min) predictions obtained by the cloudiness predictive model, when using cloudiness values estimated from the TSI images and from the prototype.

The mean absolute error between the two is, for one-step-ahead, 12.1% with a variance of 14.2%, and for 48 steps ahead, 9.9% with a variance of 10.7%, which are in accordance with the error obtained between the cloudiness estimations. These results show that an ANN image threshold model needs to be identified specifically for the images acquired by the prototype. In principle, by using cloudiness estimates from such model, the cloudiness predictions would be similar.

### 6.2. Solar Radiation

**Figure 14.**
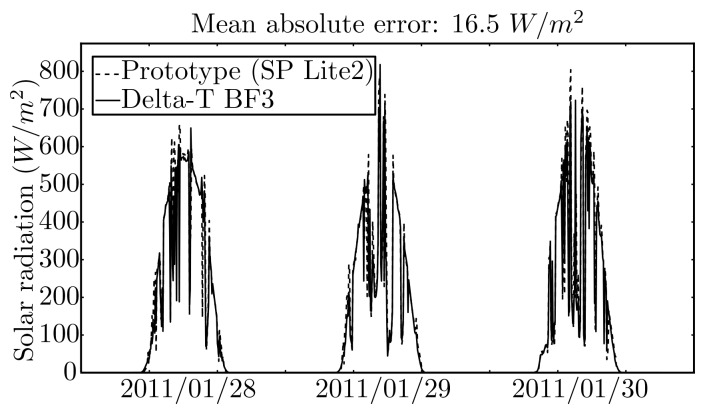
Measured solar radiation by the prototype sensor and by a weather station sensor.

The solar radiation sensor from the prototype was compared with the BF3 sensor in the weather station. Figure 14 shows the results that may be summarised by a mean absolute error of 16.5 *W*/*m*^2^, a variance of 49.2 *W*/*m*^2^, and similar dynamics, therefore showing that the pyranometer and the acquisition hardware and software are functioning properly.

**Figure 15.**
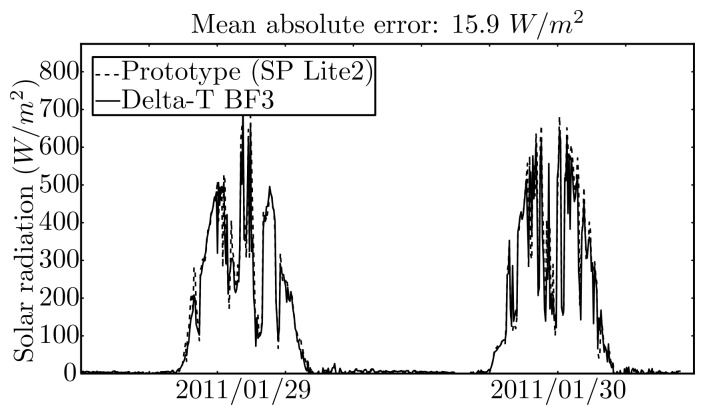
Prediction of solar radiation by the NAR model using data from the prototype and from the weather station sensor.

The solar radiation models, both NAR and 1NARX, were compared by using input data acquired by the prototype and by the weather station. In the last case the computations were carried out on a laboratory computer. For the NAR case, the mean absolute error between the two systems is 15.9 *W*/*m*^2^ for one-step-ahead with a variance of 35.5 *W*/*m*^2^, and 8.8 *W*/*m*^2^ for 48 steps ahead with a variance of 6.1 *W*/*m*^2^. For the NARX case, the mean absolute error between the two systems increased when compared with the NAR approach, *i.e.*, it is 22.4 *W*/*m*^2^ for one-step-ahead with a variance of 42.5 *W*/*m*^2^ and 15.3 *W*/*m*^2^ for 48 steps ahead with a variance of 19.4 *W*/*m*^2^, respectively. Most certainly, the increase of error was partially induced by the error propagated by the exogenous variable predictions that are employed in the NARX approach. For the case of the prototype, the propagation of error is increased by the larger error in the cloudiness estimates. Figure 15 illustrates the results for the NAR approach.

### 6.3. Atmospheric Temperature

**Figure 16.**
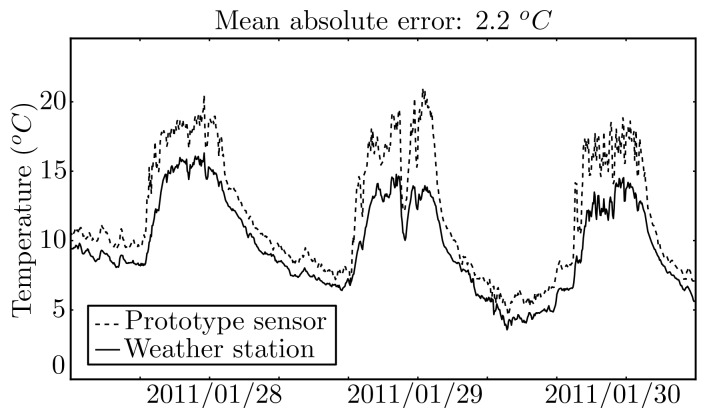
Air temperature measured by the prototype sensor and by a weather station sensor.

The comparison of the measurements made by the prototype sensor to those made by the RHT2 sensor in the weather station is illustrated in Figure 16. As it can be observed, there is an offset between the measurements, which translates into a mean absolute error of 2.2 °*C* and a variance of 1.4 °*C*. Part of this offset would probably be removed if the two sensors would have been calibrated at the same time using the same references. Another part might be explained by inappropriate housing of the sensor in the prototype, which was too small and probably providing insufficient air flow.

Regarding the NARX temperature model hav_1_ing solar radiation as exogenous input, two approaches were compared, the first using an NAR solar radiation model and the second using the NARX solar radiation model taking cloudiness as exogenous input. For both cases, the comparison was made between the temperatures measured by the prototype and by the weather station. In the first approach, the mean absolute difference was 0.1 °*C* considering one-step-ahead with a variance of 0.1 °*C* and 1.8 °*C* for 48 steps ahead with a variance of 1.5 °*C*. For the second approach, the mean absolute difference was 1.6 °*C* considering one-step-ahead with a variance of 1.5 °*C* and 1.2 °*C* for 48 steps ahead with a variance of 0.9 °*C*. These larger error values, as for the solar radiation case, are probably due to the error propagation caused by the cascade of models and due to the increased error of the cloudiness estimates generated by the prototype images.

## 7. Conclusions

A set of neural network models were identified by means of evolutionary optimisation methods, in order to provide advanced predictive functionalities to a portable cloudiness, solar radiation and air temperature prototype sensor. The results show that the estimation of cloudiness needs to be adapted specifically to the prototype acquired images. This will certainly increase the prediction accuracy of the remaining models that use cloudiness as an input, and consequently these should provide similar results as those obtained by the auto-regressive models. The implementation of models in the prototype is correct as shown by the validations, and the system is able to perform continuously to provide measurements and four-hour forecasts of cloudiness, solar radiation and air temperature. The resulting intelligent sensor is a light-weight and portable device and easy to set up on operation site. Future work will focus on complementing the implementation with a neural network trained with images acquired by the prototype, and on hardware efficiency in order to power the device with a solar panel charged battery.

## Figures and Tables

**Table 1.** MOGA parameters used for the identification of the image thresholding model.

	Parameter	Value
topology	*n* ∈ [*n_m_*, *n_M_*]	*n_m_* = 2; *n_M_* = 24
	*d* ∈ [*d_m_*, *d_M_*]	*d_m_* = 2; *d_M_* = 36
objectives	***μ****^p^*	[*ε^t^*, *ε^g^*]

MOGA	population size	100
	selective pressure	2
	crossover rate	0.7
	mutation survival rate	0.5

**Table 2.** First three lines: percent cloud cover estimation error obtained by the RFB ANN image thresholder on *hsvR*, on the reunion of the training and testing data sets, and on the validation data set. Last three lines: results obtained with the remaining methods considered, shown for comparison.

Data set	minimum	average	maximum
**X***^t^* and **X***^g^*	0.00	5.31	58.46
**X***^υ^*	0.00	4.74	43.71
**X***^t^*, **X***^g^*, and **X***^υ^*	0.00	5.22	58.46

Fix threshold	0.00	11.24	82.64
RCT method	0.00	11.34	98.21
Otsu's method	0.00	11.07	63.59

**Table 3.** MOGA parameters used in the identification of the cloudiness predictive model.

	Parameter	Value
topology	*n* ∈ [*n_m_*, *n_M_*]	*n_m_* = 4; *n_M_* = 20
	*d* ∈ [*d_m_*, *d_M_*]	*d_m_* = 2; *d_M_* = 24
prediction horizon	*ph*	48 steps (4 h)

objectives	***μ****^p^*	*ε^t^* < 8%
		*ε^g^*, minimise
	***μ****^s^*	*ε^s^*, minimise

**Table 4.** MOGA parameters used in identification of NAR and NARX global solar radiation predictive models.

	Parameter	Value
topology	*n* ∈ [*n_m_*, *n_M_*]	*n_m_* = 2; *n_M_* = 16
	*d* ∈ [*d_m_*, *d_M_*]	*d_m_* = 2; *d_M_* = 16
prediction horizon	*ph*	48 steps (4 h)

objectives	***μ****^p^*	*ε^t^* < 60 *w*/*m*^2^
		*ε^g^*, minimise
	***μ****^s^*	*ε^s^*, minimise

**Table 5.** MOGA parameters used in the identification of the NARX air temperature predictive model.

	Parameter	Value
topology	*n* ∈ [*n_m_*, *n_M_*]	*n_m_* = 2; *n_M_* = 20
	*d* ∈ [*d_m_*, *d_M_*]	*d_m_* = 2; *d_M_* = 32
prediction horizon	*ph*	48 steps (4 h)

objectives	***μ****^p^*	*ε^t^* < 0.5 °*C*
		*ε^g^*, minimise
	***μ****^s^*	*ε^s^*, minimise
