# Correction: Ferreira, P.M., *et al.* A Neural Network Based Intelligent Predictive Sensor for Cloudiness, Solar Radiation and Air Temperature.

**DOI:** 10.3390/s130709547

**Published:** 2013-07-23

**Authors:** Pedro M. Ferreira, João M. Gomes, Igor A. C. Martins, António E. Ruano

**Affiliations:** 1 Algarve Science and Technology Park, Campus de Gambelas, Pav. A5, 8005-139 Faro, Portugal; 2 Centre for Intelligent Systems, IDMEC-IST, Av. Rovisco Pais 1, 1049-001 Lisboa, Portugal; E-Mail: aruano@ualg.pt; 3 Department of Electronic and Informatics Engineering, University of Algarve, 8005-139 Faro, Portugal; E-Mails: joaomealhagomes@gmail.com (J.M.G.); rogimartins13@gmail.com (I.A.C.M.)

The authors would like to correct the acknowledgements of this article [1] as follows:
